# Development of a Web-Based 24-h Dietary Recall for a French-Canadian Population

**DOI:** 10.3390/nu8110724

**Published:** 2016-11-15

**Authors:** Simon Jacques, Simone Lemieux, Benoît Lamarche, Catherine Laramée, Louise Corneau, Annie Lapointe, Maude Tessier-Grenier, Julie Robitaille

**Affiliations:** 1 Institute of Nutrition and Functional Foods, Laval University, 2440 Hochelaga Boulevard, Quebec City, QC G1V 0A6, Canada; simon.jacques.2@ulaval.ca (S.J.); Benoit.Lamarche@fsaa.ulaval.ca (B.L.); Catherine.Laramee@fsaa.ulaval.ca (C.L.); Louise.Corneau@fsaa.ulaval.ca (L.C.); Annie.Lapointe@fsaa.ulaval.ca (A.L.); maude.tessier-grenier.1@ulaval.ca (M.T.-G.); Julie.Robitaille@fsaa.ulaval.ca (J.R.); 2 School of Nutrition, Pavillon Paul-Comtois, Laval University, 2425 rue de l’Agriculture, local 2412, Quebec City, QC G1V 0A6, Canada

**Keywords:** 24-h dietary recall, web application, Automated Multiple-Pass Method, dietary assessment, dietary intake, healthy eating index, Mediterranean score

## Abstract

Twenty-four-hour dietary recalls can provide high-quality dietary intake data, but are considered expensive, as they rely on trained professionals for both their administration and coding. The objective of this study was to develop an automated, self-administered web-based 24-h recall (R24W) for a French-Canadian population. The development of R24W was inspired by the United States Department of Agriculture (USDA) Automated Multiple-Pass Method. Questions about the context of meals/snacks were included. Toppings, sauces and spices frequently added to each food/dish were suggested systematically. A list of frequently forgotten food was also suggested. An interactive summary allows the respondent to track the progress of the questionnaire and to modify or remove food as needed. The R24W prototype was pre-tested for usability and functionality in a convenience sample of 29 subjects between the ages of 23 and 65 years, who had to complete one recall, as well as a satisfaction questionnaire. R24W includes a list of 2865 food items, distributed into 16 categories and 98 subcategories. A total of 687 recipes were created for mixed dishes, including 336 ethnic recipes. Pictures of food items illustrate up to eight servings per food item. The pre-test demonstrated that R24W is easy to complete and to understand. This new dietary assessment tool is a simple and inexpensive tool that will facilitate diet assessment of individuals in large-scale studies, but validation studies are needed prior to the utilization of the R24W.

## 1. Introduction

Accurate dietary assessment is a major concern in nutritional epidemiology because measurement error and bias likely decrease the potential to detect true associations between diet and disease [1,2,3]. Based on the findings from studies reporting good relative validity and measurement properties [4], 24-h dietary recalls (24HDRs), in which the interviewer solicits detailed information about everything the participant ate and drank from midnight to midnight the previous day, or over the past 24-h period, are now commonly used for monitoring the diets of populations and, increasingly, for studying diet and disease associations [5]. Innovative dietary assessment technologies, such as web-based 24HDRs systems, have the potential of enhancing dietary assessment through more cost-effective methods of data collection [4]. In addition to saving time and effort in processing information, the benefits of automated 24HDRs include a standardization of questions, more flexibility of completion at any time and location, an ease to obtain repeated intra-individual measures including with geographically dispersed populations, as well as an improvement in the estimation of servings consumed by the use of standardized images [6]. Moreover, the acceptability and feasibility of web-based dietary assessment methods in cohort studies have led to an increased interest in the development of web-based 24HDRs [7,8,9]. Fully automated, self-administered and web-based 24HDRs have been recently developed and have performed well in validation studies [9,10,11,12]. However, no such tool has been specifically developed to assess dietary intakes of a French-Canadian population.

In addition to the advantages related to cost-effectiveness, web-based 24HDRs offer the possibility to allow the automatic coding of food and calculation of numerous scores related to dietary and nutritional intakes. This functionality is of paramount importance to decrease the risk of error associated with manual coding of food, which is difficult to standardize between researchers. In addition, the automated calculation of scores decreases the risk of error as compared with a situation where a researcher individually computes formulae to be used with the data generated by the food recalls. It is with this in mind that an automated self-administered web-based 24-h recall (R24W) has recently been developed by our group. The R24W web application has been elaborated in order to allow the automatic calculation of comprehensive dietary intake data and dietary scores that can be generated instantly. Each food item was coded according to different nutritional criteria to enable automatic calculation of diet quality scores: The Canadian version of Healthy Eating Index (C-HEI) is a measure of diet quality that can be used to assess compliance to current Canadian dietary guidelines [13] and the Mediterranean score, as studied previously by our group [14]. The tool was developed to easily implement the calculation of additional diet quality scores and to efficiently modify the calculation of dietary scores when needed. To our knowledge, this is the first French-language fully automated self-administered web-based 24HDR that can automatically calculate dietary scores, as well as food group portions and data related to nutrient intakes. A Canadian adaptation of the American Automated Self-Administered 24-h recall (ASA24) has recently been released, but is actually only available in English [15]. Moreover, according to Thompson et al., it is desirable to adapt the food database of close-ended dietary questionnaires to the diversity of food culture in a population [16]. Therefore, the objective of this article was to describe the evidence-based development of a French-language, automated, self-administered and web-based 24HDR to be used in a French-Canadian population.

## 2. Methods

### 2.1. Automated Multiple-Pass Method (AMPM)

R24W was developed in French language and was inspired by the AMPM of the USDA, a five-step dietary interview that includes multiple passes, during which respondents receive cues to help them remember and describe foods they consumed through the 24 h of the previous day [17]. As opposed to the AMPM, which uses an open approach in the first step, R24W uses a meal-based approach. This means that the respondent has to select an eating occasion from a list, including breakfast, lunch, dinner, or snack, as a first step before reporting foods and beverages consumed during this eating occasion [18]. No limit was established regarding the number of meals and snacks that can be created and the order in which they can be entered. The following information about the context of meals and snacks was gathered: Meal name, time, location, whether the meal was eaten alone or with others, and screen-based activities during meals [19]. The integration of each step of the AMPM into R24W was informed by a series of team meetings including experts in nutrition and in dietary assessment, and by a literature review focusing on methods used to reduce the number of forgotten foods and to illustrate portions size. The R24W was programmed in such a way that the days for which the recall has to be completed are randomly generated with the possibility of using specific criteria (e.g., proportion between weekdays and weekend days). The application also allows the automatic sending of email messages to respondents prompting them to complete their food recall. Furthermore, R24W allows to automatically generate a new date when the 24HDR is not completed on the assigned date.

### 2.2. Development of the Organized Food List

An organized food list, which allows the respondent to browse that list until the selected food is found, has been developed. This food list was elaborated by a group of registered dietitians with expertise in dietary assessment. It was then expanded by consulting the Canadian Nutrient File (2010 version) database [20] and food items of other existing web-based 24HDRs [7,21,22]. To guide the selection of food to include in the R24W food list, we consulted a national publication that lists 1100 of the most commonly consumed foods in Canada [23].

The different food items were categorized according to their nutritional characteristics or the context of use during the meal. Some food items were classified into more than one category (e.g., the food item “milk” was classified in Milk/dairy products/dairy substitutes and in Beverages), so respondents can find their food as quickly and as intuitively as possible using different pathways.

Details incorporated through the food list included general food characteristics (e.g., fat percent, raw/frozen/canned), homemade vs. commercial/restaurant, and use of cooking fat. Examples of trademarks were sometimes added to food names. Food items usually associated with sauces, toppings, condiments, and seasonings are systematically linked, such that these additions are offered after the selection of the food item.

A special effort was made to represent the complexity and variety of mixed dishes in the food list. Family recipe websites that were among the first five options proposed following a Google search were consulted for guiding the selection of mixed dishes to include in the food list. Different culinary cultures were also considered when developing the mixed dishes category, given the increased popularity of such dishes in our population [24]. The food list was preliminarily validated with a sample of 20 randomly selected 3-day food records, previously collected at baseline in a nutritional intervention study conducted by our research team [25]. The 20 3-day records were completed by 10 men and 10 women between 25 years and 50 years of age. Only three mixed dishes were missing from the R24W food list out of a total of 98 mixed dishes reported in the 60 days of food records examined, suggesting that the developed food list was fairly comprehensive.

### 2.3. Search Engine

An incremental search engine was available at any time as an alternative to browsing foods by category. Each item was linked to a list of common synonyms, trademarks, and misspellings to increase search efficiency. If the list of suggestions provided was too narrow, the respondent needed to remove some words to widen the search. If the respondent still did not find the specific food item that is searched for, a food that is similar needed to be chosen.

### 2.4. Reminders and Assistance

A summary that keeps an updated, interactive list of all foods that are being reported by the respondent was included at the left-hand side of the R24W interface [22]. Edit and delete options were added for the entire meal/snack and for each particular selected food [26]. A summary showing all meals and snacks of the day was included as the last step of R24W to provide a comprehensive overview [26].

### 2.5. Portion-Size Estimation

**Figure 1:**
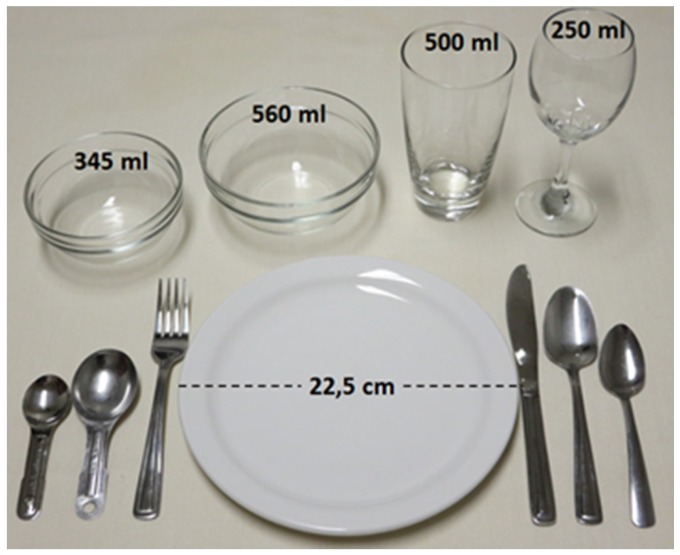
Dishes set used for the portion size photographs.

A question regarding the amount of food consumed was included after the selection of each food. Pictures of food corresponding to specific quantities were taken with a standard dish set (Figure 1) [27] in a fixed and neutral set-up to assist respondents in reporting portion size. Angled photographs were taken to simulate the average angle of viewing for an individual seated at a dining table [27]. Commercial packaging was added in addition to the serving plate, when relevant. To help determining the range of portion sizes, we consulted the most frequently chosen portion-size images of 91 food items by 966 adult participants from studies conducted at our research center [28,29,30,31,32,33,34,35,36,37], in which a web-based food-frequency questionnaire (FFQ) was completed [38]. In the FFQ, in addition to portion-size images, there was an option to select an amount that was less than the smallest portion size (“−“ option) or more than the biggest portion size (“+” option). If no participant had selected the “+” option for a particular food but many had selected the “−” option that suggested the illustrated portions were globally too big. In such a situation, we used pictures of smaller portion sizes in R24W. A specific number of pictures were associated with each food. Unshaped foods (e.g., rice, pasta, yogurt) and shaped foods (e.g., pizza, cake, cheese) were illustrated by 4 pictures. Juice and milk were represented by 8 pictures because fruit juice can be served in a glass or drunk directly from bottles of various sizes, and because milk can be consumed in large quantities as a beverage or in small quantities in coffee or tea. Single-unit foods with a single format (e.g., fresh pear) have a drop-down menu with no image. Single-unit foods with multiple formats (e.g., tortillas, muffin) are illustrated with two or three usual formats.

### 2.6. Frequently Forgotten Food

A list of foods that have been documented to be frequently forgotten was suggested after the entering of each meal and at the end of R24W [17,39]: Juice, milk, soft drink, water, and other non-alcoholic beverages; wine, beer and other alcoholic beverages; cookies, chocolate, candies, ice cream, pastries and other sweets; chips, pretzels, popcorn, mixed nuts, and other salted snacks; vegetables and fruits; cheese; bread and other baked goods; other foods/beverages.

### 2.7. Strategies for Automatic Coding

To enable automatic extraction of nutrient values, each food item was linked to a food code from the Canadian Nutrient File [20] or the USDA Nutrient Database for Standard Reference [40], when the food was not available in the Canadian Nutrient File. For mixed dishes, recipes were created by including foods from the Canadian Nutrient File using Nutrific software (Laval University, Québec City, QC, Canada) [41].

In order to allow the assessment of the overall quality of the diet, all food items were scored according to different nutritional criteria. Codes were assigned in order to enable automatic calculation of food group servings according to the Canadian Food Guide (CFG) [42] and diet quality scores: The C-HEI [13] and the Mediterranean score [14]. R24W was also programmed to calculate the probability of meeting individual nutrient requirements. Specific equations were elaborated depending on the type of dietary reference intakes (estimated average requirement, adequate intake, tolerable upper intake limit) [43].

### 2.8. Supplementary Questionnaire

R24W includes questions about factors that influence nutritional needs (sex, age, smoking, lactation, and pregnancy) [43]. Information about dietary supplements intake is also collected as it is needed to assess nutrient intakes. Finally, respondents answer questions about the representativeness of the previous 24-h intakes compared to usual eating habits as well as questions about dieting, as this information can be useful for the interpretation of the results obtained [44,45].

### 2.9. Pre-Test

A functional prototype of R24W was tested in a convenience sample to identify and address major acceptance and usability issues. Participants were recruited from an internal list of adults willing to participate in clinical studies. All individuals interested were included in the pre-test study (*n* = 29). Participants were asked to complete R24W once on an unannounced day. After the completion of R24W, a satisfaction questionnaire was sent to each participant. For each question, there was a comment box provided to give participants a chance to explain their responses and/or comments. Participants were asked about ease to access, to understand and to complete R24W, ease to remember foods consumed with the proposed sequence, ease to find foods consumed in the Web-based application, quality of portions size pictures shown and ease to select the portion size consumed using these pictures. For all questions, participants had to indicate a response on a 5-point Likert scale, ranging from “totally agree” to “totally disagree”. One of the objectives of the pre-test was to use responses and comments to these questions for developing tutorials that would help the completion of R24W. Finally, to express their satisfaction about completion time of R24W, participants were asked, “How would you describe the completion time?” The possible answers were “too short”, “too long”, “adequate”, “neutral” or “I don’t know”. This pre-test study was approved by the Clinical Research Ethics Committee of Laval University (#2014-186/30-09-2014).

## 3. Results

**Figure 2:**
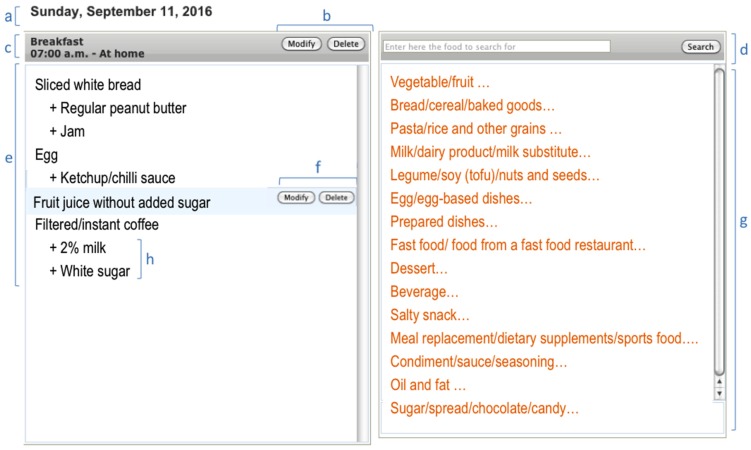
Example of a food selection screen shot from the web-based 24-h recall (R24W). (**a**) Day covered by the food recall; (**b**) Edit/Delete options for the current meal or snack; (**c**) description of the meal occasion; (**d**) search engine; (**e**) interactive summary featuring the selected food; (**f**) Edit/Delete options for a selected food item; (**g**) 16 main food categories; (**h**) selected additions to a specific food item. Note: The application is not currently available in English, but for the benefit of the reader, this figure was translated from French to English.

**Figure 3:**
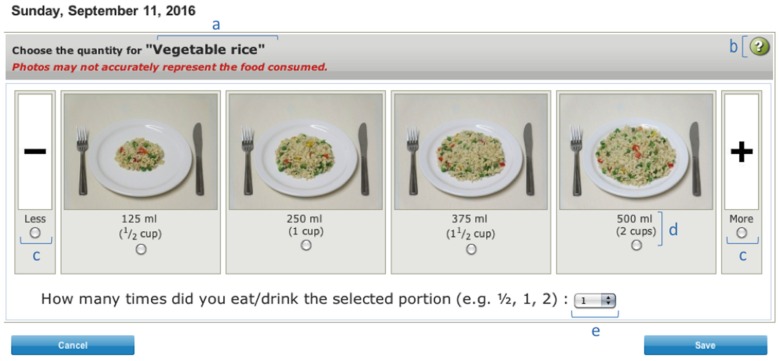
Example of a portion-size estimation screen shot from the web-based 24-h recall (R24W). (**a**) Name of the food item; (**b**) help button; (**c**) less or more options that allow respondents to choose a smaller or bigger portion size than the portion sizes at both ends; (**d**) description of the amount of food shown in the associated picture; (**e**) drop-down menu. Note: The application is not currently available in English, but for the benefit of the reader, this figure was translated from French to English.

A total of 14 months was needed to develop and pre-test the R24W. The 2865 food items were sorted into 16 main categories that was further separated into 98 subcategories (see Supplementary Materials). A total of 1491 different images depicted up to eight serving sizes per food. However, most (72.8%) of the food items are assessed with only four serving size pictures, as shown in Table 1. The main food selection screen and the portion-size estimation screen are illustrated in Figure 2 and Figure 3. From a total of 1023 mixed dishes, 687 recipes were created.

R24W does not provide any direct feedback to respondents. Researchers can extract data as a list of foods and beverages selected by the respondent; nutrient intakes; probability of adequacy of nutrient intakes; food group servings according to the CFG; C-HEI; or as the Mediterranean score. Data can be automatically extracted from a single recall, or as an average from multiple recalls and for any specific meal occasions (e.g., snacks).

### Pre-Test

Participants’ characteristics are presented in Table 2. Participants were aged from 23 to 66 years of age, had various levels of education and computer skills, and most of them were frequently involved in meal preparation.

A large majority of respondents (90%) agreed that R24W was easy to access, to understand and to complete. A total of 86.2% of the respondents agreed that the questions were logically presented and that it was easy to find the right food. A total of 89.7% of the respondents were satisfied with the quality of the images used for portion-size estimation, and agreed that these images helped them to make a choice. The time needed to complete R24W varied among the respondents. For 27.6% of the respondents, less than 20 min were needed, while 31.0% of respondents took between 20 and 30 min, 24.1% took between 30 and 45 min, 7.0% took between 45 and 60 min, whereas 10.3% were not aware of the time needed to complete R24W. That being said, 88% of the respondents found self-reported completion time to be acceptable. Comments received during the pre-test guided the development of a walkthrough video tutorial that will be mandatory on the first login in future studies using R24W.

## 4. Discussion

An automated, self-administered, web-based 24HDR (R24W) has been developed for use in a research setting, although it also has the potential to be used in clinical practice. The R24W application is based on the AMPM, but obtains information using a meal-based approach in the first step. R24W allows automatic calculation of different diet quality indicators in addition to nutrient intakes.

The choice of the AMPM to guide the development of an automated, self-administered, web-based 24HDR is consistent with other groups that used the same method for the development of similar technologies for adults or children [7,12,21,22,46,47,48]. In fact, there is growing evidence that web-based 24HDRs with AMPM-based methodology can estimate energy and/or nutrient intakes with a good level of validity in adults [9,10,11,12]. In an adult feeding study by Kirkpatrick et al., a web and AMPM-based 24HDR provided good estimation of food intakes, although a traditional 24HDR performed slightly better relatively to true intakes, items consumed but not reported and items reported but not consumed [11]. More recently, Thompson et al. found that an interviewer-administered 24HDR and a web-based 24HDR provided equivalent estimations for 87% of nutrients or food groups in an ethnically diverse adult population [9].

Instead of the open approach used in the first step of the AMPM method, R24W was developed to obtain information using a meal-based approach in the first step. In a pilot study by Subar et al., where the meal-based and the unstructured food list in a web-based 24HDR were tested and compared, adult participants expressed a strong preference for the meal-based version [18]. R24W was also designed to offer respondents the option of reporting their food using a traditional search engine or browsing food categories. In the pilot study conducted by Subar et al., respondents have expressed the need for a more flexible search engine [18]. Accordingly, to ensure the flexibility of our search engine common synonyms, trademarks, and misspelling were linked with each food item.

For most of the food items, four angled food photographs were presented simultaneously in common-sized dishes. Similarly, Zoellner et al. showed that, using a computer-based 24HDR, the replacement of standardized portions, traditionally used in conventional 24HDRs, by four pictures simultaneously resulted in increased coefficient correlation in portion-size estimation, as compared with a conventional 24HDR [49]. Although the use of food photographs and common-sized dishes is known to help estimate portion size [50,51], evidence is lacking about the best type of photographs to use in a self-administered tool. In a feeding study where the exact consumption of food was known, participants had to estimate their portion sizes using various presentations of digital images [52]. In that study, Subar et al. found that neither of the type of image (aerial vs. angled) was more accurate. In addition, they observed that respondents had a strong preference for simultaneous presentation vs. sequential presentation, as well as four vs. eight images simultaneously. The size of the photographs included in R24W is large enough to contain the dishes and to show the nature and texture of the food, as these characteristics have been described to be the most important features to be present for portion-size estimation [27].

R24W was developed as a simple and easy to understand format. This was confirmed by results obtained in the pre-test. The pre-test participants were generally well educated and interested in food. These characteristics are not necessarily representative of the usual population. However, because of these characteristics, the pre-test sample was more likely to eat a variety of food, including less common food items [53], which can be considered as an advantage for pretesting our application. The pre-test was useful to collect comments from the participants in order to develop tutorials that will help the completion of R24W in the future. The use of help screens or a tutorial has been suggested to guide all participants equally and minimize potential errors derived from their varying degrees of knowledge [6]. Therefore, several video tutorials, including a mandatory walkthrough tutorial, were developed following the pre-test study and are now included in R24W.

A major strength of R24W is that it has been developed by nutrition experts who have extensive experience in research settings and dietary assessment methodology [54]. Professional judgment has been demonstrated to be a valid resource for developing dietary assessment tools [55]. The development of R24W was also inspired by other similar technologies that had already been validated [11,12,22]. Another strength is that it is a simple and user-friendly tool, as suggested by results from the pre-test. In addition, the automatic coding of responses will provide time-saving and internal-consistency benefits to researchers that would otherwise need to enter each food item separately into specialized nutrition software to calculate nutrient intakes [10,56]. Furthermore, automatic calculation of nutrient values, dietary scores, and food group servings enables the study of associations between nutrition and health using various approaches while reducing the risk of errors associated with a decentralized approach where each researcher is in charge of performing the calculations of scores and other diet quality indices. One of the R24W limitations, just like other web-based 24HDRs, is that it cannot overcome the methodological problems inherent to self-report [4]. Individuals may still not report their food consumption accurately for various reasons related to knowledge, memory, and the interview situation [16]. Although a full replacement of an interviewer by a self-administered tool can possibly resolve some bias in dietary assessment, it could also create new ones. Advantages of the traditional dietitian-based interview are that the dietitian can directly interact with the individual when a mistake in the declared quantities is suspected, thus avoiding errors in potentially difficult portion size estimates, and can help trigger the respondent’s memory [57]. On the other hand, it has been suggested that limited contact with the interviewer tends to minimize social desirability bias [16] and, consequently, may encourage reporting of more food items [58]. The relatively small sample size of the pre-test is another limitation of this study, because it did not allow us to draw definite conclusions about acceptance or usability issues. However, it aided in fixing technical problems, improving the search engine and developing video tutorials. Finally, R24W was designed to be administered to adults only. It has been suggested that it is necessary to adapt self-administered tools when targeting children and adolescents [59,60,61,62]. As a next step, our group will conduct a study to validate R24W.

## 5. Conclusions

We described the evidence-based development of an automated, self-administered web-based French-language 24HDR that is adapted to a French-Canadian food context. It has the potential to enhance the feasibility and cost-effectiveness of collection of high-quality dietary data in large-scale studies. It is to be expected that the development of such web-based applications will facilitate the combined use of different dietary assessment methods in research settings, which has been described as a way of collecting more comprehensive dietary intake data in epidemiologic studies [63,64].

## Figures and Tables

**Table 1:** Distribution of the food items according to the number of pictures used for the portion-size estimation.

Number of Pictures Used for the Portion-Size Estimation	Proportion of Food Items (%)
0	17.7%
1	2.8%
2	3.3%
3	2.6%
4	72.8%
8	0.8%

**Table 2:** Characteristics of the sample for the pre-test (*n* = 29).

Characteristics	*n* (%)
Age (years)	46.3 ± 14.1 ^1^
16–24	1 (3)
25–44	11 (38)
45–64	13 (45)
65 or older	4 (14)
Sex	
Male	13 (45)
Female	16 (55)
Education level	
Less than high school	1 (3)
High school	6 (21)
College	9 (31)
University	13 (45)
Self-assessed computer skills	
Poor/medium	7 (24)
Good	7 (24)
Very good/excellent	15 (52)
Involvement in meal preparation	
Never	3 (10)
Rarely/sometimes	3 (10)
Often/most of the time	23 (79)

^1^ Expressed as a mean ± standard deviation (SD).

## Supplementary Materials

The following are available online at http://www.mdpi.com/2072-6643/8/11/724/s1, Table S1: Distribution of the 2865 food items in the web-based 24-h recall (R24W).
