# Supplementary material for: Development of a Web-Based 24-h Dietary Recall for a French-Canadian Population
Source: Nutrients. 2016 Nov 15;8(11):724. doi: 10.3390/nu8110724 (PMC5133109; doi:10.3390/nu8110724)
Supplement: Supplementary file 1 [file nutrients-08-00724-s001.docx]

Supplementary Materials: Development of
a Web-Based 24-h Dietary Recall for
a French-Canadian Population

Simon Jacques, Simone Lemieux, Benoît Lamarche, Catherine Laramée, Louise Corneau,
Annie Lapointe, Maude Tessier-Grenier and Julie Robitaille

**Table S1.** Distribution of the 2865 food items in the web-based 24-h recall (R24W) **^1^**.

| **Food Category** | **Number of Items** | **Item Subcategory** | **Number of Items** |
| --- | --- | --- | --- |
| **Vegetables/Fruits** | 227 | Vegetable juice | 3 |
|  |  | Vegetable | 142 |
|  |  | Fruit | 82 |
| **Bread/Cereal/ baked goods** | 308 | Bread/bagel/English muffin/tortilla | 64 |
|  |  | Breakfast cereal | 125 |
|  |  | Hot cereal | 28 |
|  |  | Bar/muffin/sweet bread | 22 |
|  |  | Crackers/croutons | 17 |
|  |  | Brioche/turnover/croissant/Danish | 8 |
|  |  | Pancake/waffle/french toast | 28 |
|  |  | Flour/breadcrumbs/wheat germ/semolina/bran | 13 |
| **Pasta/rice/other grains** | 80 | Pasta | 23 |
|  |  | Rice/other grain | 57 |
| **Milk/Dairy products/Milk substitutes** | 127 | Milk | 31 |
|  |  | Plant-based drink (soy, almond, etc.) | 6 |
|  |  | Ice cream/frozen dessert | 20 |
|  |  | Milk-based dessert | 21 |
|  |  | Cheese | 30 |
|  |  | Yogurt/Kefir | 19 |
| **Meat/Poultry/Fish and seafood** | 432 | Beef/veal/pork/lamb/horse/game | 187 |
|  |  | Poultry | 94 |
|  |  | Fish and seafood | 129 |
|  |  | Giblets (blood sausage, liver, kidney, brain, foie gras, etc.) | 4 |
|  |  | Processed meat (bacon, terrine, pâté, cold cut, etc.) | 18 |
| **Legumes/soy (tofu)/ nuts and seeds** | 100 | Legumes (bean/peas/lens) | 19 |
|  |  | Tofu/meatless product | 23 |
|  |  | Veggie spread (hummus, vegetarian patty) | 8 |
|  |  | Nuts and seeds | 41 |
|  |  | Peanut butter/almond/other nut butter (cashew/sesame, etc.) | 9 |
| **Eggs/egg-based dishes** | 65 | Egg | 11 |
|  |  | Egg-based dishes | 54 |
| **Mixed dishes** | 1023 | Salad (garden, pasta, couscous, beans, etc.) | 34 |
|  |  | Sandwich | 52 |
|  |  | Soup | 109 |
|  |  | Meat-based dishes (beef, pork, veal, lamb, etc.) | 86 |
|  |  | Poultry-based dishes | 66 |
|  |  | Fish or seafood-based dishes | 54 |
|  |  | Pasta or rice-based dishes | 236 |
|  |  | Vegetarian dishes | 42 |
|  |  | Cheese-based dishes | 8 |
|  |  | Exotic, multiethnic dishes (Asian, Indian, Italian, Mexican, etc.) | 336 |
| **Fast food** | 78 | Fries/poutine/garnished potato | 10 |
|  |  | Hamburger/hot dog | 22 |
|  |  | Pizza/turnover/calzone/nachos | 37 |
|  |  | BBQ chicken/fried chicken/chicken wings | 5 |
|  |  | Fried Chicken/Fish nuggets or fillet | 2 |
|  |  | Onion rings/garlic bread/pogo-sticks | 2 |
| **Desserts** | 103 | Donut | 8 |
|  |  | Cookie | 23 |
|  |  | Square/crumble | 6 |
|  |  | Cake/brownie/pudding | 37 |
|  |  | Fruit jelly (Jell-O^®^) | 2 |
|  |  | Pastries | 12 |
|  |  | Pie | 15 |
| **Beverages** | 75 | Alcoholic beverage | 16 |
|  |  | Hot beverage (coffee, hot chocolate, tea, herbal tea) | 31 |
|  |  | Energy drink (Red bull^®^, Guru^®^, Monster^®^, Rockstar^®^, etc.) | 3 |
|  |  | Soft drink/soda | 8 |
|  |  | Sports drink (Gatorade^®^, Powerade^®^, etc.) | 3 |
|  |  | Water/carbonated water/mineral water | 3 |
|  |  | Milkshake/smoothie/slush | 3 |
|  |  | Fruit juice | 4 |
|  |  | Flavored beverage powder/liquid | 4 |
| **Salted snacks** | 18 | Bretzel | 4 |
|  |  | Chips | 4 |
|  |  | Popcorn | 8 |
|  |  | Other salted snacks | 2 |
| **Meal substitutes/ dietary supplement/ sport nutrition products** | 20 | Bar | 3 |
|  |  | Nutritional drink | 12 |
|  |  | Energy candy/gel | 2 |
|  |  | Yeast powder | 2 |
|  |  | Protein powder | 4 |
| **Condiments/sauce/seasoning** | 101 | Broth/consommé/stock | 4 |
|  |  | Condiment | 16 |
|  |  | Salt-free seasoning/herbs (cumin, paprika, cinnamon, etc.) | 2 |
|  |  | Poultry stuffing | 2 |
|  |  | Ketchup/chutney/chili sauce | 3 |
|  |  | Mayonnaise/salad dressing | 6 |
|  |  | Pesto | 2 |
|  |  | Sauce | 35 |
|  |  | Salt/salt substitute | 5 |
|  |  | Spread (hummus, guacamole, tzatziki, etc.) | 5 |
|  |  | Dip | 2 |
|  |  | Dressing | 19 |
| **Oil and fat** | 32 | Butter | 7 |
|  |  | Oil | 16 |
|  |  | Margarine | 6 |
|  |  | Other type of fat | 3 |
| **Sugar/spread/ chocolate/candy** | 76 | Candy | 10 |
|  |  | Chocolate | 10 |
|  |  | Non-Dairy Creamer(Coffee-Mate^®^) | 6 |
|  |  | Jam/Jelly | 9 |
|  |  | Chewing gum | 2 |
|  |  | Syrup/sauce/molasses/honey | 15 |
|  |  | Sugar substitute/sweetener (aspartame, sucralose, stevia, etc.) | 6 |
|  |  | Sugar | 7 |
|  |  | Spread/icing (Nutella^®^, caramel, etc.) | 7 |
|  |  | Other candy | 4 |

**^1^** This table only classifies each subcategory in one category, although one subcategory may appear in more than one category.
